# Challenges and innovations in the surgical treatment of advanced Dupuytren disease by percutaneous needle fasciotomy: indications, limitations, and medico-legal implications

**DOI:** 10.1186/s13018-024-04844-3

**Published:** 2024-07-23

**Authors:** Giuseppe Basile, Federico Amadei, Luca Bianco Prevot, Livio Pietro Tronconi, Antonello Ciccarelli, Vittorio Bolcato, Simona Zaami

**Affiliations:** 1 IRCCS Orthopedic Institute Galeazzi, Milan, 20161 Italy; 2 Hand and Peripheral Nerve Center, COF Lanzo Hospital, Alta Valle Intelvi, Italy; 3 Maria Cecilia Hospital, Cotignola, Ravenna, Italy; 4 Department of Movement, Human and Health Sciences, University of Rome Foro Italico, Rome, 00135 Italy; 5 Milan Unit, Astolfi Associati Legal Firm, Milan, 20122 Italy; 6 Department of Anatomical, Histological, Forensic and Orthopaedic Sciences, “Sapienza” University of Rome, Rome, 00161 Italy

**Keywords:** Dupuytren disease, Dupuytren contracture surgery, Percutaneous needle fasciotomy, Percutaneous needle aponeurotomy, Medico-legal implications

## Abstract

**Background:**

Dupuytren disease, a chronic thickening and retraction of the palmar aponeurosis of the hands, may result in permanent and progressive flexion of one or more fingers. Percutaneous needle fasciotomy is a simple method that uses a hypodermic needle usually performed under local anaesthesia. The study aim was to report the postoperative results and complications using a percutaneous approach to treat Dupuytren contracture in a consecutive series of patients with advanced Dupuytren disease, also considering the relevant medico-legal implications.

**Methods:**

Retrospective multicentre study of all patients with Tubiana stage 3–4 Dupuytren contracture treated with percutaneous needle aponeurotomy, with no ultrasound assistance, from 2012 to 2022. Patient demographics, disease severity, treatment-related complications, and the incidence of recurrence were identified. An overview of therapeutic treatment options has accounted for 52 relevant sources spanning the 2007–2023 time period.

**Results:**

Overall, 41.7% (*N* = 200) of patients were females, the mean age was 72 years (60–89), the right hand was treated in 54.2% (*N* = 260) of patients. The little finger was involved in 50% of the patients. The 12 months mean PED was 9°, the mean quickDASH was 8, the mean URAM 6. Minor complications were reported in 18.7% (*N* = 90) of patients, typically skin lacerations (83.3%) with no clinical sequelae, and no major complications were reported. Recurrence occurred in 30% (*N* = 144) of patients.

**Conclusions:**

Percutaneous needle fasciotomy is safe and reliable even in patients with advanced Dupuytren disease, resulting in predictably acceptable outcome with low risk of complications.

## Background

Dupuytren’s disease is a chronic fibroproliferative disease consisting of the progressive pathological production and deposition of collagen in the palmar and digital fascia of the hand, which can cause contractures at the metacarpophalangeal and interphalangeal joints, resulting in permanent flexion and deficit of extension [1, 2].

The prevalence rate of Dupuytren disease is around 15%, it affects more men than women, and usually occurs between the ages of 50 and 70 years old. Moreover, in 65% of cases the retraction is bilateral. It has an autosomal dominant familiarity, with variable age-related penetrance [3]. Generally, Dupuytren disease is slowly progressive: it can take up to ten or more years before it reaches a level of severity that require surgery. In some cases, however, the evolution can fully unfold within months. It initially manifests itself with an abnormal thickening of the palm of the hand that, in more advanced cases, extends like a subcutaneous cord up to the fingers, usually the fourth and fifth one, making increasingly difficult the fully extension of them and determining a forced closing. This ‘closed hand’ condition, as the clinical picture progressively worsens, results in the objective inability to perform common manual activities, such as holding objects, as well as significant fine motor disability. Such a state has functional and quality-of-life repercussions in daily activities, including job performance; by the time the patient reaches retirement age, it contributes significantly to the impairment of the extremity and systemic capabilities. The specific structure of the hand affected by Dupuytren disease is the palmar aponeurosis, a thin but strong membrane, consisting of connective tissue and collagen, located under the skin, covering the underlying muscles and tendons. An overall remodelling of the connective tissue, neurovascular system, innervation, and immunological component has been described, together with the consideration of its direct contribution to chronic pain [4]. The etiopathogenesis of Dupuytren disease is still largely unclear, but family history is often present. Moreover, reported risk factors include the use of anti-epileptic drugs, excessive alcohol use, diabetes mellitus, and hyperlipidaemia [5]. The hypothesis of a pathogenic association between elevated and sustained levels of occupational exposure such as manual handling and vibration is also reported in research findings, and in few studies with a dose-response relationship [6]. The most widely used classification in clinical practice is the updated Tubiana-Michon classification, which takes into account disease severity based on the flexion angles of the finger joints, assessed by a goniometer [7]. This is useful also in assessing the need for surgery and includes:

Stage 0: healthy subject.

Stage N: nodules without finger extension deficit.

Stage I: finger extension deficit of 0–45°.

Stage II: finger extension deficit of 46–90°.

Stage III: finger extension deficit of 91–135°.

Stage IV: finger extension deficit > 135°.

Traditional therapy is fasciotomy, limited fasciectomy, total fasciectomy and dermo fasciectomy; the use of collagenase has been introduced in the last decade [8]. In recent years, a gradual reduction of more invasive techniques has been reported, with less invasive approaches often preferable to reduce post-operative risks and faster functional recovery, also considering age and comorbidities [9]. Epidemiology, age, work and recreational activities highlight the importance of identifying clinical severity in relation to patient counseling and treatment, through shared decision-making, with post-treatment recurrence risk disclosure [10]. There is currently no consensus recommending one specific approach. A 2018 review by Mella et al. 2018 highlighted the need for integrated choice, based on disease severity, patient preferences, risk of complications and recurrence, cost effectiveness, and no less importantly, surgeon skills and expertise [11]. The Italian Supreme Court has recently addressed treatment options, focusing on the surgeon’s level of expertise in traditional or minimally invasive techniques [12].

Percutaneous needle fasciotomy (PNF) or percutaneous needle aponeurotomy (PNA) is a minimally invasive treatment option for Dupuytren’s disease that has long been practised; it consists of the interruption and rupture of the palmar or palmo-digital contractures through a repeated needle-tip perforation, until the finger can be extended [1, 13–15].

This study aims to describe the outcomes of a consecutive series of patients with advanced Dupuytren contracture, treated with percutaneous needle fasciotomy approach, in terms of postoperative results, complications, and recurrence rate, looking also at the medico-legal implications.

## Materials and methods

**Fig. 1:**
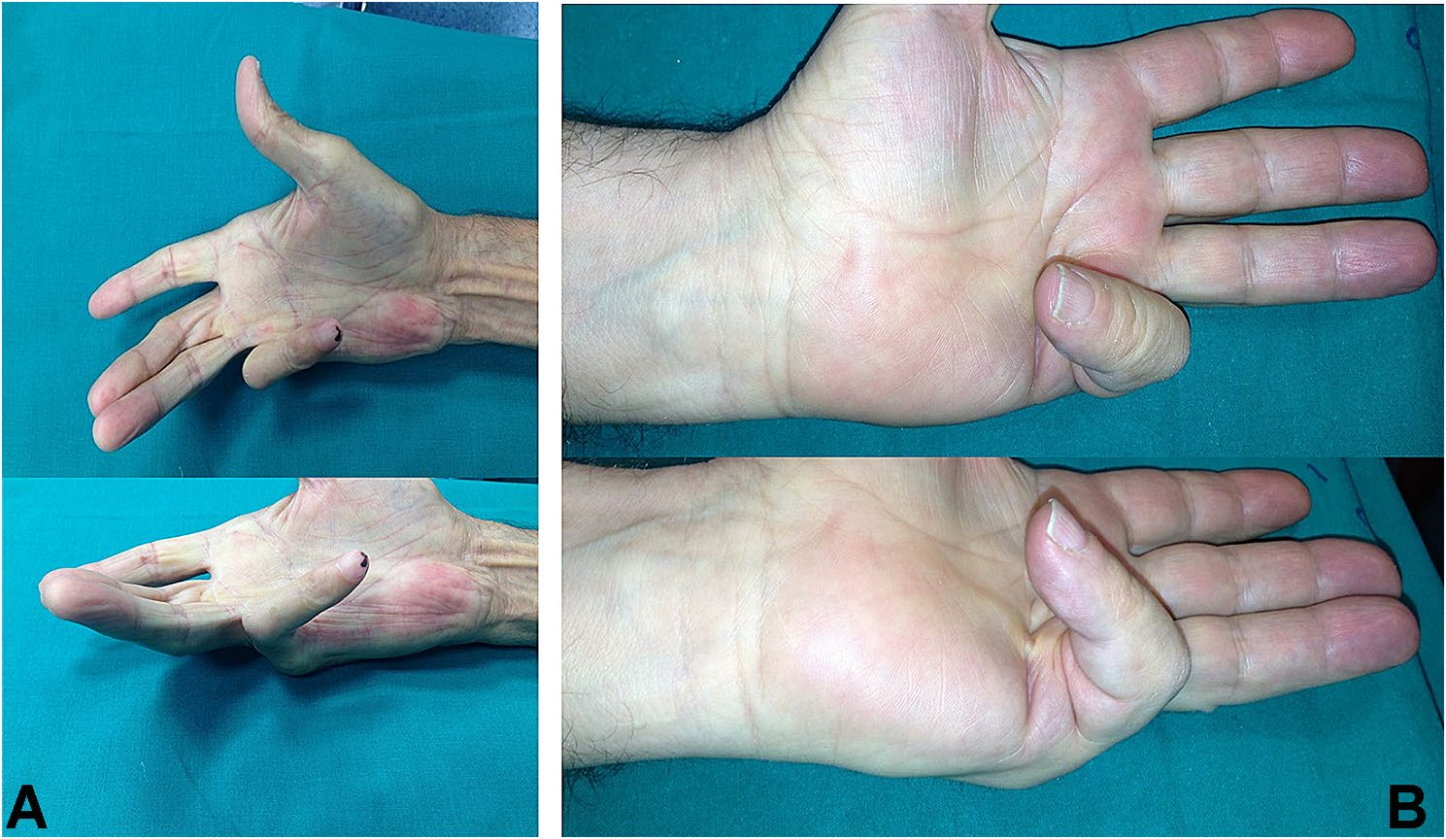
Preoperative images of Dupuytren’s disease, affecting V finger, in two different patients. **A**, Tubiana stage IV, **B** Tubiana stage III-IV

This is a retrospective multicentre study conducted by evaluating the medical records of patients who underwent percutaneous needle fasciotomy, not ultrasound-assisted, for Dupuytren disease from 1st January 2012 to 31st December 2022. All patients were informed by the orthopaedic surgeon and signed written informed consent for treatment and research purposes. A total of 480 patients were selected by the following criteria: age > 60 years, not previously surgically treated and grade III or IV according to the Tubiana classification [7, 16] (Fig. 1).

**Fig. 2:**
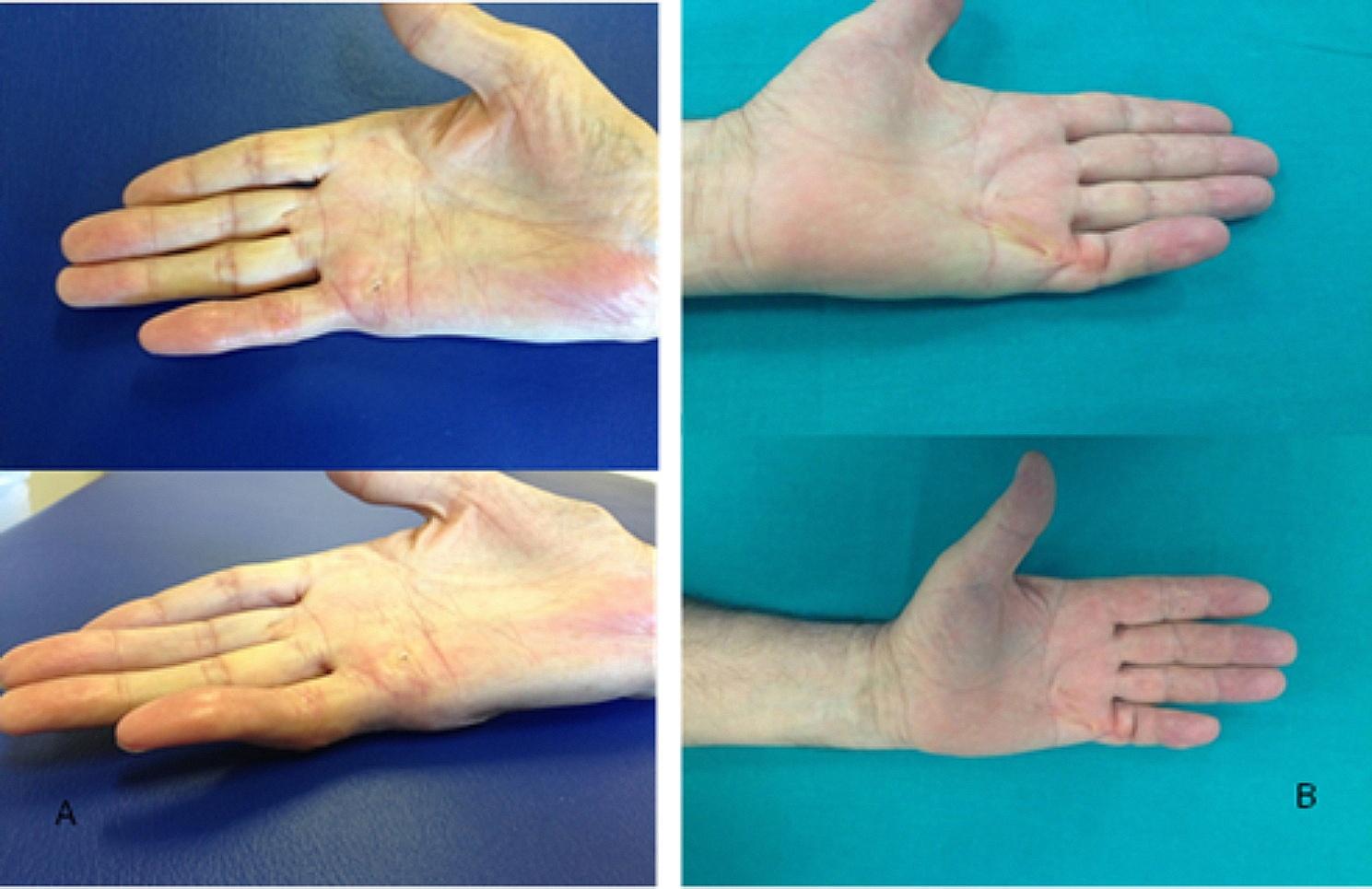
Post-surgery images of the two selected patients presented in Fig. 1. **A**, the patient selected in Fig. 1 with **A** after 1 months; **B**, the patient selected in Fig. 1 with **B** after 6 months

The age criteria reflected the indication for less invasive surgery in older patients, due to the association with higher risks of recurrence and a longer period of post-surgery inability with the other techniques [15]. In cases where more than one finger was affected, only the finger with the greatest passive extension deficit (PED) was considered, with related data collection. The overall PED of the metacarpophalangeal, proximal interphalangeal and distal interphalangeal joints and the Quick Disabilities of the Arm, Shoulder and Hand score (QuickDASH score) and the Unité Rhumatologique des Affections de la Main score (URAM score) were collected for each patient. PED measurement was performed with a standard protocol using a hand-held metal goniometer (Baseline® metal goniometer). Preoperative data were collected either at the examination for inclusion in the operating list or on the day of treatment by an experienced hand surgeon and/or occupational therapists at their respective Departments. The same professionals collected postoperative clinical data and measurements. In particular, patients were clinically assessed after surgery at 1 day, 1 week, 1 months (Fig. 2, A), 8 weeks, 6 months (Fig. 2, B) and 12 months; the PEDs, QuickDASH score and URAM score were collected preoperatively, at 1 months, at 6 months and at 12 months during follow-up examinations (Table 1). During the Sars-Cov-2 pandemic period some patients were followed up by virtual visit [17], which proved to be a valuable tool in reducing the risks of infective complications, while simplifying patient care and enhancing post-surgery monitoring. As for the surgical technique, the patient was positioned supine with the arm abducted on a couch; no tourniquet was used during the procedure. The treatment of PNF was performed under local anaesthesia without sedation. Superficial local anaesthesia was performed by superficial skin injection directly over the Dupuytren contracture with 2% carbocaine without epinephrine. The injections were carefully performed intradermally or in the upper subcutis to anaesthetise only the skin and not the digital nerves [18]. A 15- or 17-gauge hypodermic needle is inserted through the skin and subcutis. The cords must be held under tension to pull them upwards and away from deeper structures. If necessary, the process was repeated, from proximal to distal, if the cord causing residual contracture is still present. MCP and PIP joints are overcome in the surgery treatment. To avoid tendon damage, the patient was asked to actively flex and extend the finger intermittently, in order to detect the presence or absence of needle movement with active tendon excursion. The needle moving with the finger is indicative of involuntary entry into the flexor tendon sheath, requiring its repositioning. At the end of the procedure, the patient’s hand was bandaged, and the patient was informed about extremity functional rest and rehabilitation measures. A 6-week course of physio kinesitherapy was prescribed.

In the case of an overall after surgery PED of 20° or more on day one measurement and/or in the presence of a palpable cord, we considered the case to be recurrent, according to the most recent consensus reference [19, 20]. An overview of therapeutic treatment options has accounted for 52 relevant sources, spanning the 2007–2023 time period, gathered through extensive searches of medical databases Pubmed/MedLine, Cochrane Library, Scopus, Web of Science using search strings comprising the terms “Dupuytren Disease/contracture”, “fasciotomy”, “fasciectomy”, “dermo-fasciectomy”, “Collagenase injections”, “Antitumor Necrosis Factor (anti-TNF)”, “Radiotherapy”, “Focused electromagnetic high-energetic extracorporeal shockwave (ESWT)”.

## Results

Of the 480 patients who underwent percutaneous needle fasciotomy, 200 were females (41.7%) and 280 males (58.3%), while mean age at intervention was 72 years (range 60–89). At least one comorbidity was found in medical records for all the patients enrolled: arterial hypertension (78.1%, *N* = 375), diabetes mellitus (36.9%, *N* = 177), kidney failure (24%, *N* = 115) or hyper/hypothyroidism (17.9%, *N* = 85). Fifty-six patients (11.7%) were regular smokers, while no one reported habitual heavy use of alcohol (3 or more average daily alcoholic units [AU] for men and 2 or more AU for women according to World Health Organization definition). The right hand was affected and treated in 260 patients (54.2%) and the left hand in the other 220. Figure 2 illustrates the fingers affected by the disease: the little finger was involved in 240 patients (50%), the ring finger in 206 patients (43%), the middle finger in 24 patients (5%) and the index finger in 10 patients (2%). Tubiana stage III resulted in 344 patients (71.7%), IV in 136 (28.3%).

At preoperative assessment, patients had a mean total PED of 113°, a mean quickDASH score of 24 and a mean URAM score of 37. Post-surgical scoring assessments were summarized in Table 1.

**Table 1:** Preoperative and post-surgical scoring assessments

Time	Mean PED	mean quickDASH	mean URAM
Preoperative	113°	24	37
1-month post-surgery	18°	30	35
6 months post-surgery	15°	10	12
12 months post-surgery	9°	8	6

Post-surgical outcome of selected patients with preoperative images showed in Fig. 1 could be seen in Fig. 2 after 1 months for the A patient, after 6 months for the B patient.

Minor post-surgical complications were reported in 90 (18.7%) cases: in seventy-five cases (83.3%) resulted in skin lacerations, requiring bandaging, and dressing for mean 7 days, whereas oedema or a transient dysesthesia, which recover spontaneously in a mean time of 2 weeks, were reported in 15 patients (16.7%). No major complications such as nerve and flexor tendon injuries occurred in our case series. Recurrence at 12 month follow-up was found in 144 patients (30% of cases treated).

## Discussion

Dupuytren disease usually manifests as an indolent nodule on the palm of the hand, most frequently along the fourth or fifth ray. Over time, the nodule turns into a fibrous cord that tends to flex the fingers progressively and irreversibly, causing the inability to extend the finger or fingers involved. Subjects affected by advanced Dupuytren disease generally complain of functional limitation and strength deficit, forcing the subject to spare hand use with negative repercussions on psychological wellbeing and quality of life as a whole. Patients may also experience mild pain/discomfort to severe pain, although in more advanced cases, painful symptoms are less prevalent than functional ones. In this pathology, in fact, pain generally coincides with finger extension deficit. In any case, some patients complain severe pain and this symptom may be due to the presence of nerve branches into the fibrous bands or nodules, and to the overall rearrangement of the connective tissue [4, 21]. There are several classifications for the evaluation of Dupuytren disease in the literature but none, to date, has yet achieved wide consensus. The most widely used in clinical practice for surgery indication, and adopted in our retrospective study, is the aforementioned Tubiana-Michon classification [22], which grades disease severity on the flexion angles of the finger joints. In this retrospective multicentre series, the inclusion criterion of III and IV stage, with a prevalence of III stage and a mean PED of 113°, defines an overall severe condition, with a high level of impairment for those affected.

A scientific literature review focused on overall surgical techniques and complication rate reported permanent nerve injuries and flexor tendon ruptures in extremely low percentage in the Tubiana series (0.2%) and in the Foucher ones (0.05%) [23]. Van Rijssen et al. performed a prospective randomised controlled trial comparing percutaneous needle fasciotomy and limited open fasciectomy in 117 hands with a five-year follow-up [24]. For Tubiana stages I and II percutaneous fasciotomy was equal to limited fasciectomy in terms of efficacy, while for Tubiana stages III and IV limited fasciectomy resulted superior. Limited fasciectomy was, however, associated with a 5% major complications’ rate, compared to no major complications in the percutaneous needle fasciotomy group [25], but the outcome largely depends on baseline severity stage, age at surgery and patient preferred approach, and must weighed with longer follow-up time than ours. The 5-year recurrence rate in the needle fasciotomy group was 84.9%, inversely proportional with age at surgery, compared to 20.9% in the limited fasciotomy group. Badois et al. reported on a multicentre study involving 799 patients and 952 hands, a total of 3736 percutaneous needle fasciotomies, with a clinical improvement in more than 71.2% of cases at stage III and 56.6% of cases at stage IV [14, 26]. Zhou et al. compared one-year outcomes, after statistical weighting, of seventy-eight patients who underwent percutaneous needle aponeurotomy with 103 to limited fasciectomy, with low Tubiana grading (88% in stage I or II); percutaneous needle aponeurotomy was found to be linked to a lower mild complication rate. Interesting were also the findings pointing to greater patient satisfaction, better job performance and daily activities, and overall hand function in the percutaneous needle aponeurotomy group [27].

Our retrospective case series reported a 30% recurrence rate, which is similar to most other currently available findings. However, the one-year post-surgery assessment resulted in optimal scoring: mean PED of 9°, mean quickDASH of 8 and a meanURAM of 6. Another interesting finding, also for the purpose of correctly informing patients when weighing surgical options, and with respect to the post-operative course and early functional recovery, was that no major complications such as tendon injuries or nerve/vascular injuries were observed in our departments. Only limited minor complications (18.7%) were in fact reported, which usually need two-week medication at most. This can also mean good execution technique, where minor complications can be traced back as a risk linked to the same surgical aggression of the site and therefore could be only mitigated in duration. On the other hand, a less aggressive approach is associated with a slightly higher recurrence rate, but it could be safely repeated. While partial fasciectomy is mostly deemed the preferable therapeutic intervention, currently available guidelines and evidence-based findings point to potentially valuable alternative treatment avenues as well, both surgical and pharmacological/non-invasive. For the sake of thoroughness and broader contextualization, therapeutic options for Dupuytren Disease are summarized in Table 2.

**Table 2:** Succinct overview of surgical and non-surgical treatment approaches

Technique/therapeutic pathway	Treatment specifics	Indications
**Surgical Techniques**		
*Regional (or selective) fasciectomy*	It is based on the excision of the fascia that is grossly affected only (such as pretendinous cords and involved natatory ligaments in the palm and structures that are visibly affected in the fingers) [28]. Even though the disease progresses to the point of impacting the clinically normal palmar fascia, this approach has proven successful in correcting MCP joint contractures and some PIP contractures and carries an acceptably low morbidity rate. Untreated areas can still develop the condition. Usually, primary and recurrent disease can benefit from such an approach.	Even though regional fasciectomy is ineffective at preventing disease recurrence, it can achieve deformity correction and lead to faster hand function recovery compared to a large fasciotomy [29].
*Extensive (or radical) fasciectomy*	The procedure relies on the excision of the entire palmar fascia, also including tissue that looks healthy overall, in order to prevent recurrence.	Such a surgical approach, which is rather uncommon nowadays, entails a higher postoperative morbidity risk (hematoma has been reported in 14% of cases, nerve irritation or damage in 6%). An upside is the relatively low recurrence rate, reported to be around 11% [30, 31]. Patients are also exposed to a higher risk of prolonged postoperative edema and stiffness.
*Dermofasciectomy*	The procedure relies on the removal of the diseased fascia along with overlying skin. A full thickness skin graft is applied after the wound is resurfaced [32]. Two incisions (one from the distal interphalangeal joint of the affected digit to the distal palmar flexion crease, and a transverse palmar incision, to form an L shape) are needed. A selective fasciectomy is then carried out, aimed at partially closing the incision site. Surgery requires a full-thickness skin graft, harvested from the hypothenar eminence. A portion of the palm is left open, and an extension splint is applied. Splint removal occurs after 4 days, and the skin graft is applied to the palm. The palm is splinted again for 1 week.	The procedure, rather radical in nature, it is usually an option only for recurrent or severe disease. Recurrence rates are low, being similar to those of extensive fasciectomy. Among the noteworthy downsides: prolonged recovery, skin graft failure, donor site scarring, a higher complication rate, and poor skin color/texture match [32, 33].
**Non-surgical/Pharmacological Therapeutic Options**		
*Collagenase injections*	Injection into a Dupuytren cord (mostly made up of collagen) can bring about enzymatic disruption. Such an option lends itself to cases involving Dupuytren contracture with a palpable cord. The mixture of two collagenases binds, unwinds, and cleaves type I and type III collagen in the cords in a synergistic fashion, while it does not affect neurovascular structures [34]. CCH injection has a considerable degree of safety and are reportedly associated with only rare severe complications, and it especially works well on MCPJ contractures affecting the metacarpophalangeal joint (MCPJ) or low-severity manifestations. While deemed safe and minimally invasive, long-term effectiveness is lower than partial fasciectomy [35, 36].	Collagenase Clostridium histolyticum (CCH) was approved by the US Food and Drug Administration (FDA) for the treatment of Dupuytren contracture in a single digit during a 30-day treatment cycle; recommended 2 dose was 0.58 mg per injection. A 2022 systematic review [37] accounting for 3753 joints in 2675 patients has drawn the following conclusions: Initial contracture reduction was more successful with metacarpophalangeal (MCP) than Proximal Interphalangeal (PIP) joints (respectively 77% vs. 36%). A 23% recurrence rate was reported in successfully treated joints, mostly from 12 to 24 months, and at times as early as 6 months. As for treatment-related adverse effects, 94% of patients reported one or more, although most such effects were fairly minor and self-resolving (e.g., peripheral oedema, extremity pain, contusion); The rate of major surgical complications was 1%, with only two patients suffering nonsurgical complications such as tendon injury without a complete rupture and anaphylaxis.
*Corticosteroids*	These agents have anti-inflammatory properties and cause profound and varied metabolic effects. They modify the body’s immune response to diverse stimuli. Triamcinolone is used in the treatment of inflammatory dermatosis responsive to steroids. Corticosteroids can allay inflammation through the suppression of polymorphonuclear leukocytes migration and capillary permeability reversion It decreases inflammation [38].	Steroid injection may give rise to a regression of nodules and cords linked to early-stage Dupuytren. Beneficial effects have also been observed in the treatment of knuckle pads. Evidence of efficacy is still inconclusive, due to available studies lacking control groups and not enough double blinded randomized trials [39].
*Antitumor Necrosis Factor (anti-TNF)*	An ongoing phase 2 randomized controlled trial [37] has shown how the injection of an anti-TNF agent, adalimumab, directly into the nodules can lead to the downregulation of the myofibroblast phenotype, as reflected by the reduction in expression of α-SMA and type I procollagen proteins at 2 weeks, compared with saline control at 2-week follow-up. Though not yet conclusive, such findings appear to potentially pave the way for a biological therapeutic response to Dupuytren.	Intra-nodular injections of 40 mg adalimumab in 0·4 mL are reportedly effective in lowering nodule hardness and size [40].
*Radiotherapy*	Low-dose radiotherapy may halt disease progression via inhibition of myofibroblasts. Cycling cells are targeted directly. Although the exact action dynamics are still unclear, radiotherapy is thought to inhibit fibroblast proliferation and induce an anti-inflammatory effect. National Institute for Health and Care Excellence (NICE) permits the use of radiation therapy for early Dupuytren disease [41].	Research findings on the effectiveness of radiotherapy in Dupuytren are still largely inconclusive, since there is dearth of studies comparing radiotherapy to non-invasive approaches or other nonsurgical treatments [42]. Such a technique however does hold promise for the prevention of further progression and symptoms, while it cannot correct existing contractures.
*Focused electromagnetic high-energetic extracorporeal shockwave (ESWT)*	While such an approach is still under researched and experimental in nature, A recent study has hypothesized that ESWT may affect TGF-β signalling, stem-cell propagation, growth factor stimulation or modulation of pain pathways via COX2, substance P or calcitonin gene-related peptide (CRGP) [43]. A remarkable degree of pain reduction (*p* < 0.05) in the ESWT group based on visual analogue scale was reported, but the trial failed to show a statistically significant improvement of the secondary outcome parameters as patient-related outcome scores (assessed by Michigan Hand Questionnaire, DASH or URAM). The study relied on a blinded randomized trial following for 18 months an ESWT group (*n* = 27) as opposed to a placebo group (*n* = 25), all of them patients with painful Dupuytren’s nodules.	ESWT relies on acoustic waves characterized by a sharp, abrupt, and rapid pressure changes as a wave front faster than the speed of sound, followed by a longer negative tail to trigger a body response. Such a technique was first described in a 1980 study showing successful kidney stone resolution by high-energetic focused electrohydraulic ESWT. Numerous studies on various tissues point to the beneficial effects of ESWT. For instance, plantar Ledderhose’s disease of the foot sole, which is quite similar to the nodular stage of Dupuytren of the hand from a clinical and histological standpoint, nodule-derived pain can be considerably allayed by high-energy, electromagnetic-generated focused ESWT [44]. In Dupuytren disease is a therapeutic option in early stage.

In order to plan the best therapeutic pathway and to make appropriate patient selection for each procedure, it is essential to provide thorough information and consider the patient’s expectations [45]. The patient candidate for surgical treatment must be made aware of both frequent and rare risks associated with the surgical procedure.

It is worth outlining some medico-legal considerations as well, especially with regard to the highly sensitive and multifaceted aspects of clinical risk management and healthcare professional liability [46]. First of all, the timing of the surgical treatment. It should be stressed that the treatment is necessarily conservative in the first phase of the disease and in younger patients, and surgery is indicated when the condition has a recent onset; symptoms such as severe pain and overall functional disability must also be taken into account to identify the best treatment options. In fact, in case of improper untimely treatment and failure, negligence-based claims could be filed. In this case, forensic doctors and orthopaedic experts called to testify as expert witnesses always have to establish exact indications and whether all the therapeutic measures alternative to surgery have been weighed and possibly implemented. Conservative treatment associated with rehabilitative intervention in early phases of Dupuytren disease can in fact have positive effects in terms of improved hand function, delaying surgery, while also facilitating the preservation of ergonomic and proprioceptive gestures. Conservative treatment is then a valid alternative that should be explored until it is no longer viable due to unbearable pain/disconfort or severe disability. Precisely because of the possible risks associated with the surgical procedure, and particularly for some type of surgical approach for Dupuytren contracture, it is essential for orthopaedic surgeons to plan a correct therapeutic pathway, which must necessarily rely on accurate communication and information provision, all of which is to be documented as part of the informed consent process [47]. Far from being the mere provision of information, communication is key in the therapeutic alliance and for a sound doctor-patient relationship, and must also involve listening to the patient, answering questions in a comprehensible fashion, making sure that the patient has understood such information and account for their concerns, priorities and expectations. Explaining and discussing feasible alternatives is a major cornerstone of the disclosure process, with written documentation and final informed planning, as patients may not be able to assess risks in abstract terms and should therefore rely on a framework of comparison to make a truly informed decision [48]. At the same time, patient should always be able to rely on a thorough illustration of alternative options, which is the only way to properly uphold the personal right to self-determination. No less essential is the rehabilitation programme to be performed after surgery for Dupuytren’ contracture, through an effective splinting programme and/or targeted exercises designed to prevent potential complications such as wound healing disorders, oedema, and scar management, and to maintain the surgical correction and finger flexion restoration [49]. Rehabilitation programs should be directed towards restoring hand function and monitoring the development of complications that could compromise the outcome and increase recurrence rates [50]. It is essential in that regard to outline an individualised care process that also meets the patient’s need for knowledge about the disease, prognosis, treatment, and rehabilitation options [51]. It will be vital for doctors and facilities to be able to prove adherence to evidence-based guidelines and best practices with clinical documentation. Under many jurisdictions (particularly under tort statutes, in fact), should negligence-based malpractice litigation arise, the onus will be on the professionals and facilities to prove compliance with all requisite standards of care and clinically validated guidelines.

Our retrospective study had some limitations regarding the assessment of the prevalent use of the hand and the definition of work activity, which might be useful in determining the pathogenic association between hand activity and disease. In addition, longer follow-up, even considering a higher mean age at surgery, could be useful in defining recurrence rate, in order to compare the results of the different approaches more effectively, also for patient disclosure.

## Conclusions

Percutaneous needle fasciotomy has proven to be beneficial in Dupuytren disease and its short-term effectiveness is well documented and was confirmed also by our multicentre retrospective study as well. It is a simple and quick method, with a short period of work and functional inability for the patient, limited care requirements and low overall costs [52]. Recurrence rates are slightly higher, depending also on comorbidities and post-surgery rehabilitation compliance, but major complications are rarely reported. Most patients recover quickly, and the procedure is performed under local anaesthesia in day-surgery setting [53]. It can also be used in patients at an advanced disease stage with satisfactory results, possibly preventing finger amputation. Moreover, the mini-invasive procedure can be repeated with ease, also in older patients. This procedure has similar short-term outcome and patient satisfaction compared to open procedures and the use of collagenase as reported in Zhang meta-analysis [53]. Surgical treatment of advanced Dupuytren’s disease must be customised to the patient’s characteristics and needs. It is of utmost importance in that regard to provide patients with thorough information on surgical and non-surgical treatment options, complications and recurrence rates, and overall care planning and rehabilitation pathways.

## Data Availability

No datasets were generated or analysed during the current study.

## Abbreviations

PED: passive extension deficit
QuickDASH score: Quick Disabilities of the Arm, Shoulder and Hand score
URAM score: Unité Rhumatologique des Affections de la Main score
PNF: Percutaneous needle fasciotomy
PNA: percutaneous needle aponeurotomy
